# “B” Regulatory Subunits of PP2A: Their Roles in Plant Development and Stress Reactions

**DOI:** 10.3390/ijms24065147

**Published:** 2023-03-07

**Authors:** Csaba Máthé, Csongor Freytag, Adrienn Kelemen, Márta M-Hamvas, Tamás Garda

**Affiliations:** Department of Botany, Faculty of Science and Technology, University of Debrecen, H-4032 Debrecen, Hungary; freytagcsongor@gmail.com (C.F.); keleadri0613@gmail.com (A.K.); hamvas.marta@science.unideb.hu (M.M.-H.); gtamas0516@gmail.com (T.G.)

**Keywords:** protein phosphatase PP2A, B subunit, subcellular localization, metabolism, development, signal transduction, abiotic stress, biotic stress

## Abstract

Protein phosphatase PP2A is an enzyme complex consisting of C (catalytic), A (scaffold) and B (regulatory) subunits. B subunits are a large family of proteins that regulate activity, substrate specificity and subcellular localization of the holoenzyme. Knowledge on the molecular functions of PP2A in plants is less than for protein kinases, but it is rapidly increasing. B subunits are responsible for the large diversity of PP2A functioning. This paper intends to give a survey on their multiple regulatory mechanisms. Firstly, we give a short description on our current knowledge in terms of “B”-mediated regulation of metabolic pathways. Next, we present their subcellular localizations, which extend from the nucleus to the cytosol and membrane compartments. The next sections show how B subunits regulate cellular processes from mitotic division to signal transduction pathways, including hormone signaling, and then the emerging evidence for their regulatory (mostly modulatory) roles in both abiotic and biotic stress responses in plants. Knowledge on these issues should be increased in the near future, since it contributes to a better understanding of how plant cells work, it may have agricultural applications, and it may have new insights into how vascular plants including crops face diverse environmental challenges.

## 1. Introduction

The present review intends to give novel insights into the functioning of a family of eukaryotic regulatory proteins in plants. These are the heterogenous “B” subunits of protein phosphatases PP2A. As we will see, these subunits have various functions in plant cells, and in the upcoming years, much knowledge should be acquired on the mechanisms by which they control subcellular and whole-plant events from the regulation of cell cycle and development to plant stress responses.

Reversible protein phosphorylation is a crucial type of post-translational modification [1,2]. PP2A is a PPP (phosphoprotein phosphatase) type of serine–threonine protein phosphatase. It is highly abundant in all eukaryotes, which is valid for plants as well [3,4]. PP2A holoenzyme is a heterotrimer consisting of the scaffolding “A” subunits, the regulatory “B” subunits and the catalytic “C” subunits. “A” and especially “B” are protein families consisting of multiple isoforms with tissue- and cell-compartment-dependent expression patterns. This variability is responsible for the multiple ways of localization and substrate usage of the holoenzyme. The “C” catalytic subunit has a much smaller number of isoforms than for the other two subunits and a definitely much smaller, than the number of protein kinase isoforms. For example, Arabidopsis and tomato have only five PP2A/C variants, while in potato, there are six, and in alfalfa, at least three isoforms [5,6,7,8].

PP2A controls a large number of key cellular metabolic and regulatory processes. Functioning of a significant number of proteins, including enzymes that are key players of metabolic pathways, depends on their phosphorylation state. Examples for important PP2A functions in plants include [1,8]: (i) the regulation of primary and secondary metabolism; (ii) regulation of polar auxin transport by the PIN proteins [9,10,11]; (iii) regulation of key signaling pathways including those mediated by brassinosteroids (BRs) and ABA and hence of plant development from embryogenesis to the morphogenesis of vegetative organs—PP2A exerts this function along with other phosphatases; (iv) although we still have little knowledge on the regulation of the cell cycle by PP2A, a significant number of publications has recently emerged on this topic [10]; (v) they are involved in the responses of plants to abiotic and biotic stresses and in immune responses against bacteria and fungi or resistance against aphids [12,13,14].

Since most of the studies on PP2A were performed in Arabidopsis, here we give a brief presentation of its PP2A/C subunits. C1, C2 and C5 catalytic subunits belong to subfamily I and have important functions in the regulation of stress responses, e.g., oxidative stress [6,15,16]. C3 and C4 subunits belong to subfamily II [17]. Some of their functions include: (i) the organization of microtubule (MT) structures—cortical microtubules (CMTs), the preprophase band (PPB), mitotic spindle and phragmoplast; (ii) the regulation of polar auxin transport by dephosphorylation of PIN auxin efflux carriers in the embryos and axial organs, which influence their developmental patterning; (iii) the light-dependent regulation of nitrate reductase [18,19,20].

As we stated before, B subunits are responsible for the multiple localizations and functions of PP2A complexes. Moreover, if a single-substrate protein contains multiple phosphoserine/threonine dephosphorylation sites, they can modulate the overall catalytic activity of the complex and determine which sites will be modified and which amino acids will remain unchanged [4]. B subunits (PP2A/B) have at least 17 isoforms in Arabidopsis and probably more in soybean. For example, 43 Arabidopsis and 83 soybean genes for B″ were identified, and for soybean, many of them were associated with salt/drought stress [6,8]. As in other eukaryotes, in plants (judging mainly on the basis of studies on Arabidopsis), they are further subdivided into B (B55/PR55), B′ (B56/PR61, 54–74 kDa) and B″ (PR48/PR72/PR130, 72–130 kDa) with a wide range of molecular weights (54–130 kDa), while not much is known about the B‴ (PR93/PR110) subunits for plants [3,6,21,22]. These are subfamilies with multiple members, and there is minimal homology between the subfamilies. For plants, the following members were proven to occur: B/α, β, B′/α, β, δ, ε, κ, η (B′η is a subfamily with the following members: γ, ζ, η, θ) and B″/β, γ, EMB40, FASS/TON2, GDO [6,21]. The α and β subunits of the B subfamily show 43–48 amino acid sequence homology with their yeast and animal counterparts [6]. For plants, at least the B″ subunits were shown to contain the ASBD1 and ASBD2 domains necessary for binding the PP2A/A-C dimer in eukaryotes [21]

As we will see in this review, the TON2/FASS protein of Arabidopsis is a B″ subunit with one of the widest ranges of function among PP2A/B proteins [20,23]. The *ton2/fass* mutants have dysfunctional or absent PP2A/B″(FASS) subunits. The homozygote recessive phenotypes show severe developmental disorders (incomplete differentiation of axial organs), and their meristematic cells lack the PPB of MTs. PPB is important in the organization of the mitotic division plane in plants. FASS is known to interact with C3 and C4 catalytic subunit isoforms [17,20,23]. Dwarfism and altered developmental phenotype (e.g., defects in the development of axial organs) have been known for a long time for *fass* mutants [24].

The main objective of the present review is a detailed presentation of the role and functioning of B subunits in plant life with insights into the current knowledge gaps and future research directions in the field. Accordingly, we will critically survey their involvement in the regulation of metabolism (Section 2.2), of cell functioning/signal transduction, whole-plant development (Section 2.3) and of abiotic/biotic stress reactions (Section 2.4). Although “A” scaffolding subunits are very important in the functioning of PP2A holoenzymes as well, they are not the subject of this review. For a start, we will overview the multiple subcellular localizations of the multiple members of the PP2A/B subunit family.

## 2. Roles and Functioning of B Subunits

### 2.1. Subcellular Localizations of B Subunits

As for the other eukaryotes, diverse members of the B subunit subfamilies have diverse subcellular localizations in plants. This is largely dependent on the nature of targeting/signal amino acid sequences of the given protein [25]. This diversity of localization is shown in Table 1. Localization is in a close relationship with the target/substrate; thus, there is functional significance of a given PP2A holoenzyme complex, as we will see in the subsequent subsections.

TON2/FASS is perhaps the most well-known B subunit studied in terms of subcellular localization in Arabidopsis. Its gene is expressed ubiquitously in the Arabidopsis plant [23]. This B″ subunit is part of a protein complex called TTP (Ton1-TRM/Ton2 recruiting motif-PP2A). TRM and Ton1 show sequence homologies with animal centrosomal proteins [20,43]. In this complex, FASS binds both A (all three isoforms) and C (C3, C4) subunits and binds the holoenzyme to Ton1/TRM, which is important in recruiting the PP2A holoenzyme to cortical microtubules (CMTs) and to the preprophase band (PPB) by a mechanism described in detail by Spinner et al. [20], Schaefer et al. [44] and Rasmussen and Bellinger [45]. TRM is a multiprotein family where several members (typically, TRM1) bind MTs, while others do not [43]. Other B″ subunits are also known to bind at least the A subunit of holoenzymes [46]. In light of these statements, GFP-Ton1 was localized to the radial walls/membranes of Arabidopsis root apical meristem cells [47]. Besides FASS, Ton1 interacts with RCN1, a PP2A/A subunit known to be important in the targeting of PP2A to PIN1, a polar auxin transporter. Given that the PP2A/C3 and C4 subunits interact with both FASS and PIN1 [18,20], it is possible that these B″ subunits play a role in auxin transport. However, FASS was not localized to the same membrane site, which raises the possibility that Ton1 interacts with other B subunits as well [48].

Regarding MTs, FASS shows a weak interaction with katanin, an MT-severing enzyme in the conical cells of Arabidopsis petals [49].

Localization of B subunits at the chromatin level is known for B′α and B′β, which localize to the centromeres at the initial stages of meiosis I and meiosis II [31]. They are important in sister chromatid cohesion as described in Section 2.3.

All the above data referring to Arabidopsis and subcellular localizations are given in Table 1. Waadt et al. [37] and Durian et al. [13] presented the locations for the B subunits in Arabidopsis as follows: cytoplasm—Bα, β, B′—all known subunits, B″α, β, γ, δ; nucleus—Bα, β, B′α, β, ε, ζ, η, θ, κ, B″α, β, γ, δ; nucleolus—B′ζ, η, θ, κ; mitochondria—B′ζ; peroxisomes—B′θ. Since then, other localizations (e.g., plasma membrane) for B subunits have been found, as specified in Table 1.

### 2.2. Regulation of Plant Metabolism by B Subunits

**Figure 1:**
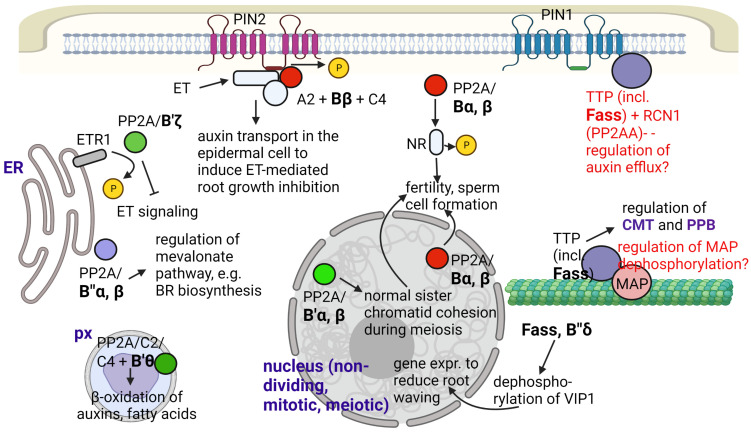
Summary for the known and supposed roles of PP2A/B subunits in Arabidopsis metabolism and development. Abbreviations: CMT—cortical microtubule; ET—ethylene; ETR1—ethylene response 1; NR—nitrate reductase; PM—plasma membrane; PPB—preprophase band; px—peroxisome; TTP—Ton1-TRM-PP2A complex; VIP1—VirE2-interacting protein 1. Functions that are not fully proven are indicated with red characters. Figure created with BioRender.com (accessed on 15 January 2023).

Although the metabolic regulatory role of PP2A always influences key developmental and/or stress defense events (see Section 2.3), we dedicated a separate subsection to several important metabolic pathways (see Figure 1 as well).

One of the most important metabolic changes induced by B subunits were observed for Bα and Bβ in Arabidopsis, where they interact with nitrate reductase (NR). NR is dephosphorylated, thereby activated at a phospho-Ser residue under light conditions, in a photosynthesis-dependent way. These B subunits are important in targeting PP2A holoenzymes to NR [26]. Since NR is of crucial importance for nitrogen/amino acid metabolism, it is not surprising that impairment in the functioning of Bα/Bβ will have dramatic consequences in whole-plant development, including decrease in fertility [26,27].

B′θ subunit targets the C2 or C5 subunits containing PP2A holoenzymes to the peroxisome by its PTS1 targeting signal and regulates peroxisomal β-oxidation of auxins (of indole-3-butyric acid to the more active IAA) and of fatty acids [16]. Therefore, this subunit is potentially essential for the regulation of many hormone signaling and stress response events.

B″α and B″β subunits regulate 3-hydroxy-3-methylglutaryl CoA reductase (HMGR), a key enzyme of the mevalonate pathway under optimal and stress conditions in Arabidopsis. The mechanism is discussed in [42]: Under optimal conditions, B″β promotes dephosphorylation and thereby inactivation of HMGR. In contrast, during salt stress, B″α will promote an initial decrease, then an increase in HMGR activity through transcriptional regulation of its expression, and it is involved in the reduction of root growth in mild salt concentrations. HMGR catalyzes the third step of the mevalonate pathway to produce mevalonic acid, a compound used thereafter for the production of isopentenyl diphosphate for terpenoid (including isoprenoid) biosynthesis. This pathway provides sterols (for membrane components and brassinosteroid biosynthesis), phytoalexins and other important compounds in the plant cell, necessary for both developmental regulation and stress defense.

We will discuss several important B-subunit-regulated metabolic pathways during stress responses in Section 2.4.

### 2.3. Regulation of Cell Cycle/division and Whole-Plant Development by B Subunits

The roles of “B” subunits in plant metabolism and development are summarized in Figure 1 and Table 1.

In terms of mitosis regulation, one of the most well-studied PP2A/B subunits is TON2/FASS of Arabidopsis and its homologues in other species. This is a B″ subunit that targets the holoenzyme to CMTs and PPB via the aforementioned Ton1-TRM complex [20,44,45]. This colocalization of PP2A with MTs is important in the regulation of their de novo polymerization, stability and bundling [10,50]. Ton1 has some homology with proteins that build up human centrosomes, and it interacts with Centrin, a component of Arabidopsis microtubule organizing centers (MTOC) [47]. On the other hand, MT organization depends on microtubule-associated proteins (MAPs), whose functioning depends on their binding to this cytoskeletal element [10]. For example, AtMAP65-1 is a MAP that induces non-mitotic and mitotic MT bundling only when a PP2A complex dephosphorylates it [50]. For the future, it would be interesting to see whether the phosphorylation state of AtMAP65-1 depends on FASS. To summarize the effects of FASS on the above mentioned two types of MT arrays: (i) regarding CMT, it has been known for a long time that at least part of the *fass* mutant lines is characterized by defects in the formation of normal CMT arrays [51]. Later, it was demonstrated that a lack of functional FASS decreases CMT density, leading to altered shapes of trichomes, epidermal pavement cells and hypocotyl cells (see Kirik et al., 2012 [52], for an example). (ii) As we mentioned before, the lack of functional FASS protein leads to the absence of PPBs in premitotic cells, which may alter division planes, leading to abnormal development of axial organs [52]. If FASS is present, but several TRM isoforms (that most probably link the PP2A holoenzyme to MTs) are not functional (the *trm678* mutant of Arabidopsis), PPB will be absent, but division plane anomalies as severe as in some *fass* mutants cannot be detected. The authors concluded that TRMs regulate the accuracy of division orientation but have only a partial effect on the gross regulation of division plane position [44]. However, another possible explanation is that different TRM variants have different affinities to MTs; for example, TRM1 is known to induce cell shape alteration and may bind more strongly to cytoskeletal elements. Meanwhile, there is no evidence for the binding of FASS to mitotic spindles or phragmoplasts. However, discordia1/alternative discordia1, maize proteins homologous to FASS, regulate phragmoplast orientation during the formation of stomatal subsidiary cells in the shoot epidermis [53]. Since FASS regulates MT organization both in dividing and non-dividing cells, its functioning regulates proper cell division patterning in the embryos and roots of Arabidopsis. *fass* mutants with a strongly altered phenotype such as *fass-5* are characterized by altered early embryo development and altered cellular patterning (i.e., wrong cell division planes) in the root apical meristem [20,52].

The rice retinoblastoma-related protein (OsRBR1) is dephosphorylated by a complex containing a Type II PP2A/C subunit and a B″ subunit. Phosphorylation of B″ by a CDK increases its affinity to the PP2A holoenzyme, increasing dephosphorylation of OsRBR1. This is thought to be a mechanism that contributes to the inactivation of OsRBR1 during the cell cycle [54].

Other B subunits are important for the formation and normal development of gametophytes in Arabidopsis. Bα and Bβ are important in the normal division of microspores, thereby regulating pollen fertility [28]. The B′α and B′β subunits play a key role in sister chromatid cohesion during meiosis, with the double mutants being semisterile. These two subunits play a redundant role in fertility [27,31]. The mechanism: these subunits are probably responsible for the targeting of the PP2A complex to SYN1 (after its binding to SGO1/shugoshin), a component of the cohesion complex in meiotic Arabidopsis cells. This targeting protects SYN1 from phosphorylation-mediated destabilization and consequently degradation [31].

Several B subunits are known to regulate signaling pathways that affect whole-plant development. Bβ activates the PP2A/C4+A2 complex in the presence of ethylene. This complex will remain bound to the auxin efflux carrier PIN2/EIR1 to mediate its dephosphorylation and hence auxin transport alongside the root epidermis to cause ethylene-induced root growth inhibition [29]. B′ζ is proposed to target the PP2A complex to CTR1 (constitutive triple response 1), a kinase [39]. This is a negative regulator of ethylene signaling. Binding of ethylene to its receptor (e.g., ETR1, located in the ER membrane) will render CTR1 to be phosphorylated and unstable, and this will finally trigger ethylene-mediated signaling. However, it is proposed that the B′ζ containing PP2A holoenzyme dephosphorylates and stabilizes CTR1, which will dimerize and rebind to ETR1 to block ethylene signaling and to maintain normal seedling development in Arabidopsis [39].

**Figure 2:**
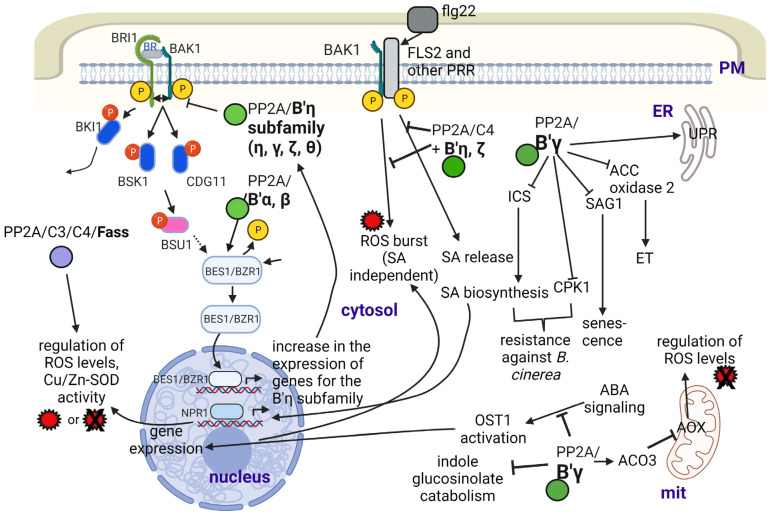
The roles of PP2A/B subunits in the Arabidopsis responses to abiotic and biotic stresses. Abbreviations: ACO3—aconitase 3; AOX—alternative oxidase; BAK1—brassinosteroid insensitive 1–associated kinase 1; BES1—BRI1–EMS–suppressor 1; BRI1—brassinosteroid insensitive 1; BZR1—brassinazole resistant 1; CPK1—calcium-dependent protein kinase 1; ET—ethylene; flg22—flagellin; FLS2—flagellin sensing receptor 2; ICS—isochorismate synthase; mit—mitochondrion; NPR1—nonexpresser of PR1; OST1—open stomata 1; PM—plasma membrane; PRR—pattern recognition receptor; ROS—reactive oxygen species; SA—salicylic acid; SAG1—senescence associated gene 1; SOD—superoxide dismutase; UPR—unfolded protein response. Figure created with BioRender.com (accessed on 15 January 2023).

B′ subunits can regulate brassinosteroid (BR) signaling in diverse and complex ways ([32]; Figure 2). Members of the B′η family target PP2A to BRI1, the brassinosteroid receptor. Upon promoting its dephosphorylation, they will prevent the formation of the BRI1-BAK heterodimer and block BR signaling. This signaling pathway will promote the expression of B′η; thus, this is a negative feedback mechanism that blocks excessive signaling and maintains normal growth of Arabidopsis [32]. On the other hand, B′α and B′β target PP2A to BZR1 to be dephosphorylated, which thus can act as a transcription factor to switch on BR-responsive genes [32,33].

Several B/B′ subunits regulate flowering time in Arabidopsis, and the way of regulation is dependent on the type of B subunit associated with PP2A [26]. Knockout mutants of Bα and Bβ were characterized by early flowering by an unknown mechanism. Meanwhile, B′γ inhibits FLC (flowering locus C) expression. The repression of FLC during vernalization is necessary for the induction of flowering integrators (FT and SOC1); therefore, this subunit seems to be a positive regulator of flowering. Since this work was based on gene expression studies, we still do not have enough data for the relevant study of PP2A holoenzyme activity in this case.

An interesting study has revealed that low levels of B′φ transcripts are necessary for the proper formation of mycorrhizal associations in tomato roots to maintain normal plant development [55].

### 2.4. Regulation of Plant Responses to Abiotic and Biotic Stresses

Many aspects of PP2A-related stress responses in plants are related to oxidative stress. This involves the production and subcellular effects of reactive oxygen species (ROS) as well as their scavenging. We and others have already reviewed many important aspects of this issue [13,14]. Therefore, in this section, we will concentrate mainly on novel insights on the B subunit - oxidative (abiotic and biotic) stress relationship (see Figure 2 and Table 1 for a summary). Firstly, we give examples to abiotic stress responses.

Regarding the B′γ subunit, aconitase 3, known to be (down)regulated by it, is involved in the tolerance against mitochondrial dysfunction during UV-B or antimycin-A stress [56]. We provide more details on this subunit in relation to its roles in the functioning in plant metabolism during biotic stresses/plant pathogen defense. B′γ may regulate plant responses to heat stress as well [57,58].

OST1 (open stomata 1) is an SnRK2-type protein kinase involved in ABA signaling that will trigger the expression of ABA-related genes, including RBOH (respiratory burst oxidase homologue, NADPH_2_ oxidase) and generates ROS. The activity of this kinase is inhibited by PP2A with the involvement of the B′θ subunit [14,59,60]. Other authors have pointed out that the above relationship is not so simple, since whether the PP2A holoenzyme works as a positive or negative regulator of ABA signaling depends on the species or organ/cell type (e.g., stomatal opening vs. root development) [37]. ABA induces the expression of the alfalfa Bβ subunit as well, but the functional implications are yet to be clarified [5].

FASS is known to be a B″ subunit involved mostly in microtubule organization to control cell shape and the cell division plane (See Section 2.3). Based on protein-protein interaction, PCR analysis and microscopy studies of GFP-fusion proteins, FASS, together with subunit B″δ is supposed to regulate, thus activates/targets a basic leucine zipper transcription factor, VIP1 to the nucleus by dephosphorylation. This will activate genes that serve in the defense of roots against mechanical and hypo-osmotic stress by reducing its waving during these conditions in Arabidopsis (Figure 1). Both types of B″ subunits have Ca^2+^ binding EF-hand motifs that help their interaction with VIP1 [40]. A recent study [41] has revealed that FASS also regulates oxidative stress responses, since it: (i) maintains normal levels of reactive oxygen species (ROS) both in meristems and differentiated root tissues; (ii) together with its interacting catalytic subunit partners (C3 and C4), it controls ROS scavenging by regulating superoxide dismutase (Fe-SOD and Cu/Zn-SOD) activities; (iii) it regulates the histone H2AX phosphorylation state. Phosphorylation of this histone variant is important for DNA damage repair by inducing the loss of nucleosomes to make DNA available for repair enzymes [61]. Much research is needed to reveal the subcellular locations and exact mechanisms of the above events.

Soybean transcriptome data showed that in this plant, genes for 26 members of the B″ family were responsive to drought/salt tolerance [62]. A B″ subunit from wheat (TaPP2AbB″-γ) was found to be localized in the plasma membrane, cytosol and nucleus. Its transcript level increased during salt, drought, cold and ABA treatments, and its overexpression in Arabidopsis seedlings increased abiotic stress tolerance [63].

Concerning biotic stresses, the excellent paper of Durian et al. [13] gives a survey on the roles of reversible protein phosphorylation in plant immune reactions, featuring PP2A. It gives an excellent insight into the relevant mechanisms where B subunits are known to be involved. His general statement is that the levels of at least the C2 and C5 Arabidopsis catalytic subunits increase during biotic stress but decrease during abiotic stress. As concerning B subunits, B′ζ, η, θ and B″α transcripts follow this trend, but for B′α, β, γ and δ, transcript levels increase during abiotic stress as well. During bacterial (*Pseudomonas syringae*) attack, the receptor-like kinase FLS2 (flagellin sensing receptor 2) binds to BAK1 (a co-receptor of the BR receptor BRI1). This complex will form a PRR (pattern-recognition receptor) system. PP2A/C4 bound to B′η and ζ is a negative regulator of this complex by dephosphorylating and thus inactivating it; hence, it inhibits PTI (pathogen/PAMP-triggered immunity). PTI should lead to the activation of plasma membrane NADPH oxidase as well as to intracellular salicylic acid (SA) release and ROS burst (see [38] and Figure 2). In general, all members of the B′η family investigated to date (γ, ζ, η, θ) are negative regulators of PTI. Part of them involve the control of salicylic acid (SA) and jasmonic acid (JA) signaling/regulation of oxidative status of the cell by regulating ROS production and by often triggering its enzymatic scavenging [12,13]. B′γ and ζ are also negative regulators of resistance to aphids, but for B′γ, this depends on light conditions. Negative regulation is true under high irradiance, but the opposite occurs at moderate light conditions [34,35]. We can assume that these mechanisms are largely dependent on environmental conditions such as temperature, light and humidity. For example, B′γ is an activator of PP2A in general, but under certain conditions, this subunit is inactivated at pathogen attack; thus, mechanisms triggering the immune responses will be activated. On the other hand, B′γ activates mitochondrial alternative oxidases (AOX, important ROS scavengers) via interacting with cytoplasmic aconitase3 (ACO3) (presumably activating its dephosphorylation at Ser91) and blocking its inhibitory effects on AOX. We do not have proof on its activation effect on the PP2A holoenzyme in this case. Basic information of the relevant effects of B subunits can be seen in the works of [15,34,35,64,65,66,67]. Excellent reviews summarizing earlier studies on cellular and molecular mechanisms are also available [13,14].

More recently, the study of Durian et al. [12] gave a complex view about how cytoplasmic PP2A complexes bearing B′γ interfere with PR induced by the necrotrophic fungus *Botrytis cinerea* and with leaf senescence. Mutant analysis and protein interaction studies showed that B′γ inhibits SA-dependent and SA-independent pathogenesis response/resistance (PR expression) and delays SA-dependent and -independent developmental leaf senescence (inhibits premature aging of leaves). The mechanisms are as follows ([12,68]; see Figure 2): (i) it interacts with and inhibits the activity of CPK1, a calcium-dependent protein kinase involved in signaling toward defense- and senescence-related gene expression in an SA-independent way; (ii) it interacts with and inhibits activity of SAG1, a cysteine protease related to senescence in an SA-independent way; (iii) it inhibits the activity of the isochorismate synthase ICS1, a key enzyme in SA biosynthesis, and it inhibits SA signaling via the positive regulator NPR1 (nonexpresser of PR genes 1).

PP2A/B′γ is also involved in ethylene biosynthesis and signaling. Li et al. [36] found that ACC2 oxidase is controlled by reversible phosphorylation with a role (presumably downregulation) of this subunit. This has been related to ethylene-triggered control of pathogen defense (see [14]).

Yeast two-hybrid assays indicate that the AvrE superfamily of type III effectors (T3Es) of several plant pathogenic bacteria (e.g., *Pseudomonas syringae* pv. tomato, *Erwinia amylovora*, *Pectobacterium carotovorum*) suppress PAMP-triggered immunity (PTI) via activating PP2A, a mechanism that will inhibit plant immune responses, including those induced by SA [69]. According to a possible model, PP2A subsequently dephosphorylates, thereby activating ORM proteins that are negative regulators of the sphingolipid pathway necessary for a hypersensitive response [70]. It was proposed that several Arabidopsis B′ subunit isoforms are required for the functionality of the PP2A holoenzyme in this context [71].

The catabolism of glucosinolates, secondary metabolites characteristic for Brassicaceae, is regulated by PP2A, with the involvement of B′γ as follows. Indole glucosinolate catabolism is under the control of indole glucosinolate methyl transferases, which is found to interact with B′γ that inhibits this activity [72]. The other two enzymes are subject to reversible phosphorylation under the control of the same subunit. These are myrosinase TGG1 and thiocyanate methyltransferase 1 [36]. Glucosinolate catabolism generates compounds important for the resistance of plants against pathogens [73].

To add to the multiple functions of B′γ, it also downregulates immune reactions by triggering a signaling pathway that leads to the dephosphorylation of calreticulin 1 (CRT1), an ER-localized chaperone that activates its protein unfolding activity [66].

## 3. Concluding Remarks

As shown in this review, the “B” regulatory subunits are uniquitous in the plant cell; their different subfamilies can be found from within the plasma membrane to the endomembrane compartments, mitochondria, nucleus and cytosol, and subunits such as FASS are of particular importance for the organization and functionality of the microtubular cytoskeleton (Table 1; Figure 1). The multiple subcellular localizations underline the main message of this paper: B subunits have many crucial functions in the plant cell. They have a wide variety of key regulatory functions from the regulation of basic metabolic processes and cell cycle regulation to basic hormone-regulated signaling pathways and to whole plant development. Furthermore, increasing evidence is proving their importance in the regulation of plant responses to abiotic and biotic stresses. A single member of this subunit family may have multiple functions. A prominent example is B′γ, with a wide variety of functions (especially, regulation of stress defense) in Arabidopsis. FASS is a B″ subunit that functions both in the regulation of mitotic and non-mitotic cytoskeletal organization and in oxidative stress responses. In addition, multiple B subunits can function in the same process. For example, unrelated members of the B′ subfamily have opposite functions in BR signaling (See Figure 2).

Despite the rapidly increasing knowledge in this field, there are many aspects that are partially and totally unknown. For example, in the past several years, FASS has proven to have more functions than previously expected. There is increasing evidence showing that besides regulating mitotic and non-mitotic microtubule organization, it plays a role in the control of oxidative stress responses, and future research is needed to answer the question: does it play a role in the control of defense against pathogens? This issue may have practical applications, since it can help in the breeding of crop cultivars with modified functioning of “B” subunits involved in biotic stress responses to increase their resistance. A challenging question: can we state unequivocally that FASS plays a role in the regulation of PIN phosphorylation and thus auxin transport? Such questions can be raised for many other types of B subunits: further research will certainly give more information on their various subcellular localizations and functions.

## Figures and Tables

**Table 1:** Summary of principal data for the most important PP2A/B subunits in Arabidopsis.

Subunit Family	Subunit Name	Subcellular Localization	Catalytic Subunit Partner	Function/Mechanism of Action	References
B	Bα, Bβ	cytosol, nucleus	unknown for Bα	male gametophyte development interacts with nitrate reductase to activate (dephosphorylate) it and promotes fertility; delay of flowering	[13,26,27,28,29,30]
	Bβ	plasma membrane-bound?	C4	ethylene-induced root growth inhibition	[29];
B′	B′α, B′β	nucleus, cytosol	C5?	sister chromatid cohesion during meiosis; dephosphorylation of BZR1 and promoting BR signaling	[6,31,32,33]
	B′γ, δ, ε, ζ, κ, θ, η	nucleus, nucleolus, cytosol	not known for all of them (C4 for B′η, ζ)	interaction of PP2A with BRI1 to dephosphorylate and inactivate it; therefore, they negatively regulate brassinolide signaling	[13,25,32,33]
	B′γ	cytosol, nucleus, plasma membrane	not studied here	promotion of flowering; modulation of PR, e.g., against *Botrytis cinerea*; regulation of resistance against aphid fecundity; inhibition of premature leaf senescence	[12,13,30,34,35,36]
	B′ζ, η	cytoplasm, nucleus, nucleolus, mitochondria; for η, plasma membrane as well	C4	inactivates the PRR system BAK1-Flg22 to reduce plant immune response	[25,33,37,38]
	B′ζ	mitochondria, cytosol	C4	dephosphorylation of CTR1 to prevent ethylene signaling; regulation of resistance against aphid fecundity;	[13,25,35,39]
	B′θ	nucleus, cytosol, peroxisomes	C2, C5	β-oxidation of IBA and fatty acids; ABA signaling via the OST1 (SnRK2)	[15,16,25]
B″	TON2/FASS	CMTs, PPB, cytosol	C3, C4	regulation of MT assembly in mitotic and non-mitotic cells; control of ROS levels, Cu/Zn SOD activity and phosphorylation of histone H2AX; dephosphorylation of the VIP1 bZIP TF for the defense against mechanical stress in roots	[20,40,41]
	B″α, B″β	cytosol, nucleus	not studied here	regulation of the mevalonate pathway under normal and stress conditions to influence isoprenoid/plant hormone metabolism	[13,42]
	B″δ	cytosol?	not studied here	dephosphorylation of the VIP1 bZIP TF for the defense against mechanical stress in roots	[40]

Abbreviations: BR—brassinolide; BRI1—brassinosteroid insensitive 1; BZR1—brassinazole resistant; PR—pathogenesis response; ROS—reactive oxygen species.

## Data Availability

This is a review article and no new data were created.
